# Atlantoaxial Subluxation due to an Os Odontoideum in an Achondroplastic Adult: Report of a Case and Review of the Literature

**DOI:** 10.1155/2015/142586

**Published:** 2015-11-26

**Authors:** Abolfazl Rahimizadeh, Housain F. Soufiani, Valiolah Hassani, Ava Rahimizadeh

**Affiliations:** Department of Spinal Surgery and Department of Anesthesiology, Pars Advanced and Minimally Invasive Manners Research Center (PAMIM), Pars Hospital Affiliated to Iran University of Medical Sciences, Tehran 1415944911, Iran

## Abstract

The authors report the first example of an adult achondroplastic dwarf with progressive quadriparesis secondary to atlantoaxial subluxation as a consequence of an os odontoideum. Actually, craniocervical region is a frequent site of compression and myelopathy in achondroplasia particularly in children as a result of small foramen magnum and hypertrophied opisthion. Moreover, very rarely in achondroplastic patients, coexistence of atlantoaxial instability as the sequel of os odontoideum can result in further compression of the already compromised cervicomedullary neural tissues, the scenario that has been reported only in five achondroplastic children. Herein, a 39-year-old achondroplastic male suffering such an extremely rare combination is presented. With C1-C2 screw rod instrumentation, atlas arch laminectomy, limited suboccipital craniectomy, and release of dural fibrous bands, reduction, decompression, and stabilization could be achieved properly resulting in steady but progressive recovery.

## 1. Introduction

Achondroplasia is an autosomal-dominant condition characterized by dwarfism, macrocephaly, rhizomelic shortening of the extremities, saddle nose deformity, and trident hand all resulting from a defect in endochondral bone formation [1, 2]. Os odontoideum is an anomaly defined as an ossicle with smooth circumscribed margins with no osseous continuity with the body of C2 [3–5]. Both achondroplasia and os odontoideum can cause canal compromise at cervicomedullary junction but with completely different mechanisms. Cervicomedullary dysfunction in achondroplasia is as a result of progressive narrowing of the foramen magnum and is mostly seen in childhood [6–9]. But cervicomedullary compression in os odontoideum is usually diagnosed in the second to fourth decades of life and is due to atlantoaxial instability mostly with forward displacement of atlas on axis and rarely with its backward subluxation [4–6, 10–12]. However, combination of these two pathologies is an extremely rare event and since the report of Gulati and Rout in 1974, only four more children with this association have been published so far [13–16].

Herein, a 39-year-old achondroplastic male with severe quadriparesis secondary to atlantoaxial dislocation due to an os odontoideum is presented. He was successfully managed with C1-C2 screw rod instrumentation and cervicomedullary decompression. To the best of our knowledge, this is the first adult example with this association in the literature. Further, in this report, we will describe the similarities and the dissimilarities between the clinical picture and the surgical strategies in these two different pathologies, in isolation and in their coexistence.

## 2. Case Report

**Figure 1:**
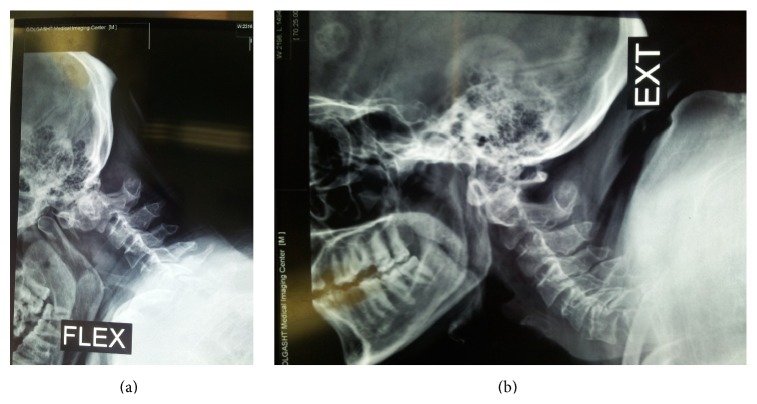
((a) and (b)) Dynamic lateral cervical radiographs showing atlantoaxial dislocation.

**Figure 2:**
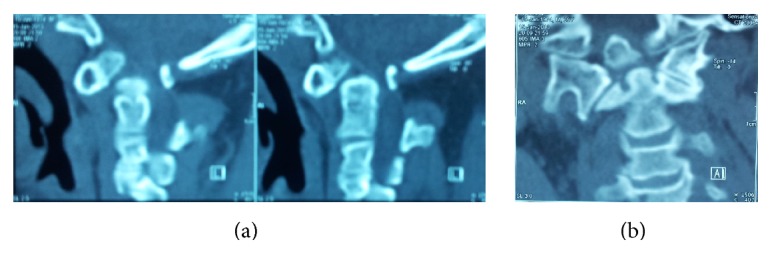
((a) and (b)) Sagittal and coronal reconstructed CT scan demonstrates os odontoideum and subluxation of C1 on C2.

**Figure 3:**
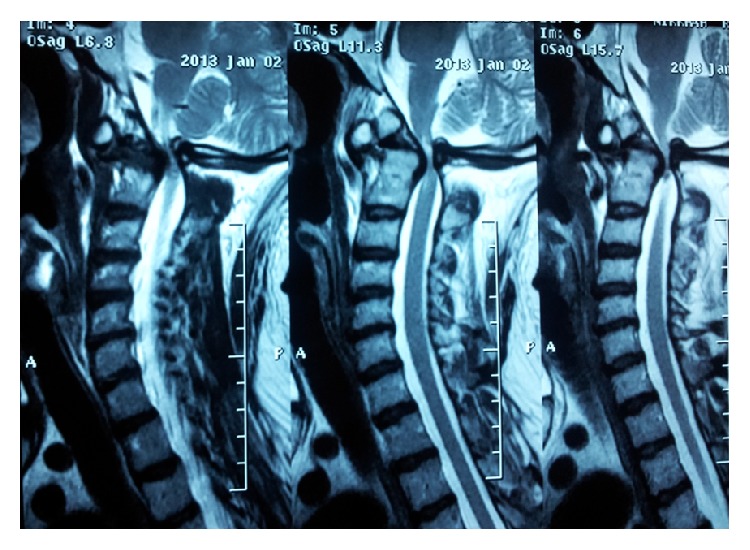
T1-weighted sagittal MR images showing os odontoideum, severe narrowing, and a hyperintense signal at cervicomedullary junction indicating myelopathy.

This 39-year-old achondroplastic dwarf man was admitted with a 9-month history of progressive weakness of all his extremities following an insignificant fall from the stairs. Before this event, his developmental landmarks and intelligence had been normal. On examination, an incontinent and bedridden dwarf even dependent in feeding was noticed. His general weakness was graded 1/5 on the right and 0/5 on the left. His tendon reflexes were increased with bilateral extensor planter responses. MRI which was done as the first diagnostic method showed an os odontoideum with marked forward displacement of C1 in relation to C2 resulting in severe stenosis at cervicomedullary junction, accompanied with a hyperintense signal in T2-weighted images indicating myelopathy (Figure 1). Lateral dynamic cervical radiographs taken two months after the fall revealed atlantoaxial dislocation with marked instability, a large head with small posterior fossa also noted (Figure 2). Axial and reconstructed CT images demonstrated dysplastic os odontoideum accompanied with anterior dislocation of C1 on C2. Forward extension of the posterior edge of foramen magnum and mild hypertrophy of the opisthion were also noted (Figures 3(a) and 3(b)).

**Figure 4:**
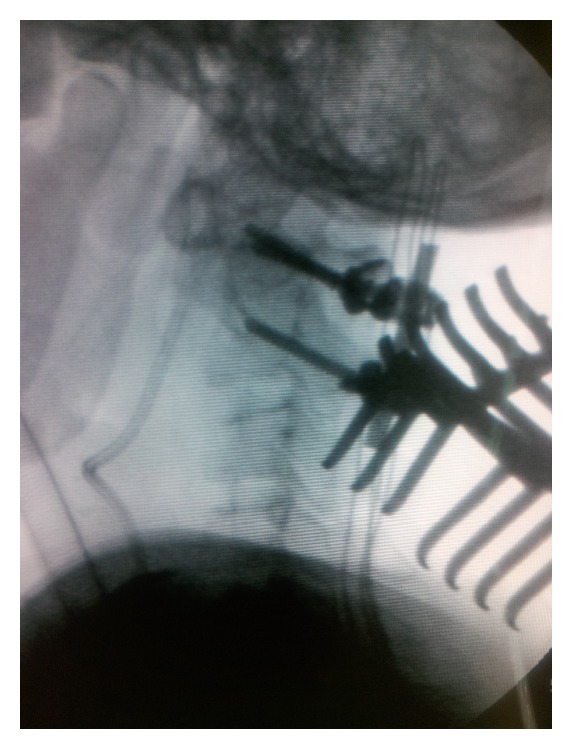
Intraoperative fluoroscopy showing alignment after posterior instrumentation.

**Figure 5:**
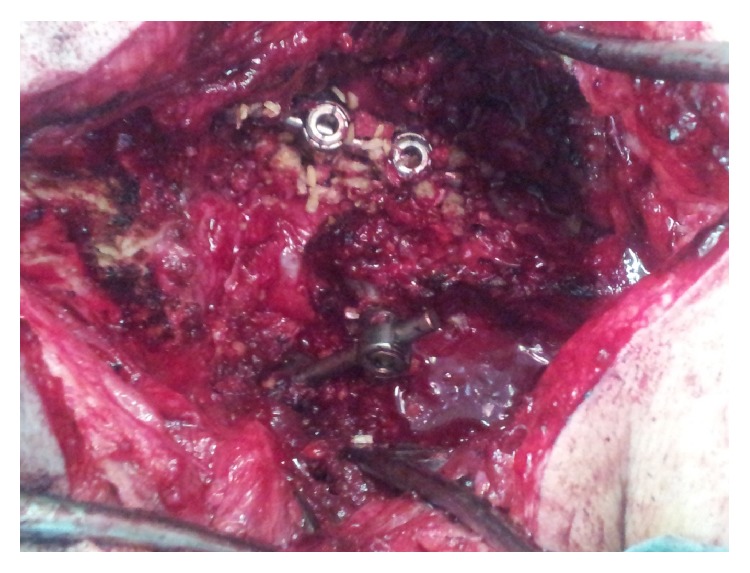
Intraoperative view showing C1 lateral mass C2 pedicle on the right and C1 lateral mass translaminar C2 on the left side.

Surgery with atlantoaxial posterior instrumentation was decided. Subsequent to placement of screws in lateral mass of atlas, C2 pedicle was inserted on the right side. But on the left side, because of overriding vertebral artery translaminar screw was done. Later, after C1 arch laminectomy, the rods were placed and the nuts were tightened in extension. Optimal alignment could be achieved confirmed with fluoroscopy (Figure 4). However, because of the faint dural pulsation at the scene and constriction of the dura by the posterior lip of occiput as well as the presence of several dense fibrous bands, a limited suboccipital craniectomy and division of the fibrous bands were accomplished. This additional procedure was followed with significant expansion of the dura and good pulsation (Figure 5).

**Figure 6:**
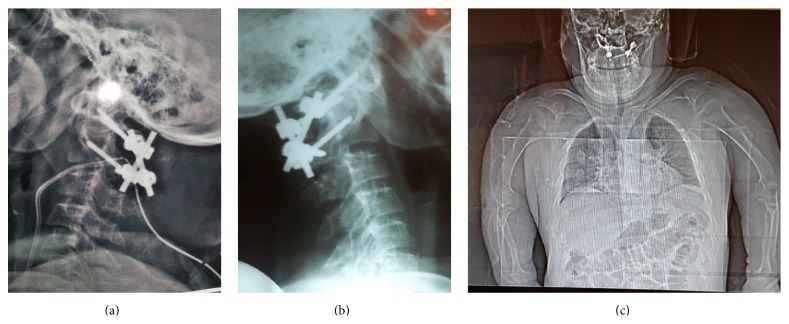
(a) and (b) Lateral cervical radiographs a day after surgery and a month after surgery. (c) AP radiographs showing this achondroplastic patient with instrumentation at C1-C2 region, note rhizomelic upper extremity and short statue of the patient.

**Figure 7:**
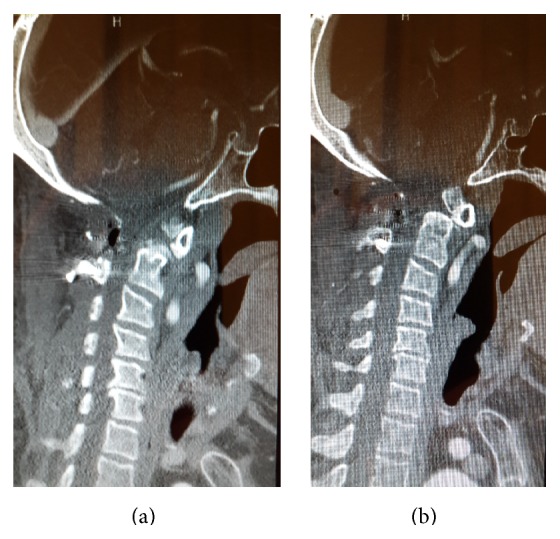
Sagittal reconstructed CT plus angiography, showing optimal alignment, and decompression of cervicomedullary junction achieved with C1 arch laminectomy and limited occipital laminectomy. Note shortness of clivus, low Trochlor Herophili, and low external occipital protuberance and integrity of the basilar artery.

**Figure 8:**
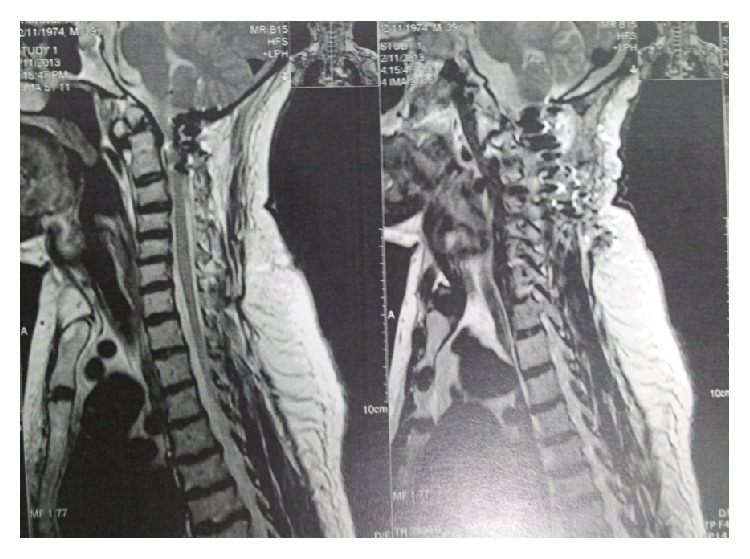
T1-weighted sagittal MR images showing alignment and decompression of craniocervical junction. Note short clivus, low Trochler Herophili, and small posterior fossa.

Postoperative course was uneventful and was followed by steady neurological recovery of the patient. Plain radiographs revealed proper reduction of atlantoaxial dislocation (Figures 6(a), 6(b), and 6(c)). Reconstructed CT angiography revealed optimal alignment of the atlas on axis, accuracy of decompression, and hopefully the integrity of the arteries of posterior circulation which we were worried about (Figure 7). MR images done two months after surgery revealed successful alignment and decompression of the cervicomedullary region (Figure 8). Now 18 months postoperatively, the patient has shown fair recovery. Now, the force on the right side is 3/5 and 1/5 on the left. He can feed himself with a spoon and is able to stand and walk a few steps with walker.

## 3. Discussion

Achondroplasia is the most common form of human short-limbed dwarfism and is one of a spectrum of diseases caused by mutations in the Fibroblast Growth Factor Receptor 3 (FGFR3) gene [1, 2].

The condition is inherited as an autosomal-dominant trait but 80% of patients are the result of new mutations [1, 2].

Os odontoideum or separate odontoid is defined as a round, ossicular remnant of the dens that is not fused to the body of the C-2 vertebra [3–5]. Whether the os odontoideum is a developmental anomaly or an acquired lesion is debated in the literature [3, 5, 17–24]. Nonetheless, in achondroplasia, narrow foramen magnum is due to defective enchondral ossification in the basiocciput [6–8].

The signs and symptoms of both os odontoideum and achondroplasia at craniovertebral junction encompass a wide spectrum but their clinical picture is classified in two main groups, symptomatic and asymptomatic [1, 2, 6–8].

Many subjects with os odontoideum remain asymptomatic throughout their life and might be only discovered incidentally [25–27]. On the other hand, in achondroplasia, where about one-third of patients show imaging evidence of narrow foramen magnum with diameters smaller than average, only 75% of these patients become symptomatic with the clinical picture of cervicomedullary compression [2, 6, 8].

In symptomatic patients affected by these two pathologies, the clinical picture of cervicomedullary compression is caused in different ways. In achondroplasia, cervicomedullary compression is the consequence of the shortening of the skull base and clivus presented as abnormally small posterior fossa, a horizontally oriented posterior squama of the occipital bone with indenting effect due to its forward extension, thickening of the posterior lip of the foramen magnum, and the thick bands of connective tissue constricting the already compromised neural structures [1, 2, 6–8]. However, in os odontoideum, cervicomedullary compromise is due to atlantoaxial instability [4, 10–12].

Diagnosis of cervicomedullary compression due to os odntoideum is mostly done in the second to fourth decades of life, although it might become symptomatic and detected in the elderly [3–5, 11, 28]. Cervical and suboccipital pain might be the first complaint of a patient with unstable os odontoideum. By far, cervicomedullary compression secondary to atlantoaxial dislocation as a consequence of an os odontoideum will eventually result in progressive quadriparesis [3–5, 10–12, 28]. Vascular insufficiency varying from simple vertigo to massive cerebellopontine infarction may rarely complicate an unstable os odontoideum which is due to torsion of the vertebral artery [29–32]. Sleep apnea may infrequently complicate this pathology.

On the other hand, most of the achondroplastic patients presented with cervicomedullary compromise are children where its presentation in adulthood should not be regarded infrequent [1–9]. In infants and young achondroplastic children, sleep apnea and sudden death are the most common features of cervicomedullary compression, whereas in older children and in adults with achondroplasia, progressive quadriparesis is the cardinal feature of cervicomedullary myelopathy [1, 2, 6, 7]. Neck pain and occipital neuralgia have been also described in achondroplasia.

In both pathologies, minor trauma plays an important role on this critical area and may cause rapid deterioration of neurological function in an asymptomatic patient with danger of serious consequences and even death [33, 34].

Plain standard and transoral cervical radiographs might show the round ossicle which is a sufficient clue for the diagnosis of os odontoideum and lateral flexion-extension radiographs can provide valuable information regarding the instability as well as reducibility of an os odontoideum [3–5, 11]. In achondroplasia, short clivus, low external occipital protuberance, hypertrophy of the opisthion, and forward extension of squamous portion of occipital bone might be depicted in good plain radiographs [1, 2, 7–9].

Reconstructed CT images are of great value in showing details of the pathology and establishment of diagnosis in these two diseases [5]. Os odontoideum is demonstrated as a round or oval ossicle instead of the dense of the axis [4, 5, 28], where reduced dimensions of the foramen magnum, the osseous overgrowth of the posterior squama of occipital bone, and forward extension of the posterior lip of foramen magnum designated as horizontalization are cardinal CT features of achondroplasia [2, 35].

Once these two pathologies become symptomatic, MRI becomes the modality of choice. Effacement of subarachnoid spaces at cervicomedullary junction and abnormal intrinsic cord signal intensity are cardinal MRI features. However, in both diseases either in isolation or in combination, dynamic MRI is of greater value since it can highlight the cord compression more precisely and demonstrate the impairment of the CSF flow more properly than static MRI [36].

In both pathologies, indication for prophylactic surgery in asymptomatic patients is controversial.

Generally, in an asymptomatic subject with a stable os odontoideum and in majority of asymptomatic achondroplastic patients with insignificant narrowing of foramen magnum, observation suffices. However, the patients or the relatives should be informed adequately of the risk inherent in conservative management.

However, surgery is strongly advocated in asymptomatic patients harboring os odontoideum with apparent atlantoaxial instability where permanent damage of the spinal cord and even death in response to relatively minor to moderate intensity traumas have been documented by some authors [27, 34]. The same is true in achondroplasia, where prophylactic surgery has been warranted in asymptomatic subjects in the presence of marked and impressive imaging features of the foramen magnum narrowing as well as with exhibition of hyperintense signal intensity at the cervicomedullary junction [37, 38]. Apparently, in achondroplastic infants and children, posterior fossa decompression diminishes the risk of sudden death and respiratory complications.

Nonetheless, the indication of surgery in symptomatic cases both in os odontoideum and in achondroplasia is straight forward. In achondroplasia, where progressive quadriparesis and documented sleep apnea both due to cervicomedullary compromise are the absolute surgical indications in achondroplasia, appearance of cervicomedullary myelopathy and vascular insufficiency are the indications of surgery in os odontoideum [4–7, 37, 38].

In achondroplasia, the procedure typically consists of suboccipital craniectomy and C-1 arch laminectomy and release of dense fibrotic bands where duraplasty remains optional. Craniectomy should be limited to approximately 3 cm in order to avoid complications such as cerebellar sagging and craniocervical instability [37, 38].

Notably, the surgical strategy in os odontoideum depends on reducibility of atlantoaxial subluxation determined in dynamic lateral radiographs. However, besides flexion-extension X-rays, skull traction is necessary in order to differentiate reducible from irreducible cases and for final assessment of reducibility especially after anesthesia [28, 39]. In reducible atlantoaxial dislocations, surgical management is crystal clear. In such instances, posterior stabilization either through posterior wiring techniques or through posterior screw instrumentation is justified [40–44]. The latter can be done via Magrel transarticular C2-C1 screw or C1-C2 screw fixation of Harms technique [28, 43, 44]. In atlantoaxial dislocation secondary to os odontoideum C2 translaminar screw can be used as an alternative option of C2 pedicle screw [45].

Irreducible atlantoaxial dislocation secondary to os odontoideum is a considerable challenge for the surgeons. Nowadays and in modern era release of C1-C2 facet joints is preferred to odontoidectomy for reduction of atlantoaxial subluxation due to os odontoideum [46–50]. Corresponding facet release can be accomplished either anteriorly or posteriorly. The corridor for anterior release of the facet joints has changed from transoral route to retropharyngeal and recently to endoscopic endonasal route [46–50]. Posterior release of the facet joints can be easily done with or without C2 nerve root resection [51]. The posterior rotating rod strategy introduced by Chang-Wei et al. remains another option in irreducible cases which can be done after facet release [52]. Reduction via horizontal screw rod construct is another promising technique for reduction described recently [53]. Nonetheless, reduction as well as stabilization depends on the surgeon's expertise.

## 4. Odontoidectomy

Removal of the os odontoideum to get rid of its persistent compressive effect on the cervicomedullary region has always been an option in irreducible conditions. This can be done through the transoral-transpharyngeal, transnasal, and transcervical corridors. Via these techniques, the odontoid, the coexisting offending anterior bony masses, redundant ligaments, granulation, and hypertrophic scar tissues should be removed till adequate ventral decompression can be achieved.

Transoral odontoidectomy for os odontoideum dates back to 1968, when Greenberg et al. accomplished this procedure in two patients with IAAD due to hypoplastic separate odontoid successfully [54] in the largest series; 28 out of 134 patients operated by Menezes for os odontoideum had irreducible atlantoaxial dislocation with cervicomedullary compromise in whom a transoral decompressive procedure was performed [55]. For several years, this technique was recommended as the best option and remained the procedure of choice for irreducible conditions affecting the atlantoaxial joints for many authors.

However, because of the drawbacks and morbidities encountered in the transoral-transpharyngeal corridor such as local infection, retropharyngeal abscess, meningitis, palatal dehiscence, pharyngeal dehiscence, delayed pharyngeal bleeding, velopalatine incompetence, and persistent hoarseness, the transnasal endoscopic odontoidectomy was introduced.

Leng et al. described endonasal endoscopic resection of os odontoideum with fewer complications in comparison to the former routes [56]. Magrini et al. described an endoscopic endonasal odontoidectomy in a patient with Down syndrome suffering irreducible IAAD due to os odontoideum [57]. Visocchi et al. in 2011 described their experience with endoscopic assisted microsurgical odontoidectomy in 7 patients where one of them had os odontoideum [58]. In 2011, Gempt et al. used the same procedure in a 52-year-old female with os odontoideum [59]. Recently, Yen et al. operated on 13 cases suffering from IAAD via transnasal endoscopic odontoidectomy without resection of nasal turbinates. Two out of these 13 cases had os odontoideum [60].

A much safer route is endoscopic odontoidectomy via transcervical corridor that has been described by Wolinsky et al. [61]. This route is the same as has been described by Smith and Robinson where the only difference is that the incision is at the high cervical region [62]. However, in fixed dislocation due to os odontoideum, mainly of the dystopic variety, the cervicomedullary compromise may not necessarily and mainly be from the os odontoideum but the assimilated atlas or the body of axis might be the cause of further compression. Therefore, resection of the caudal part of clivus, arch of atlas, and the cranial part of axis might be necessary.

Regardless of the corridor used for resection of the os odontoideum and other offending tissues, instability of the atlantoaxial joints exists and might become even worse. Therefore, after achievement of adequate decompression for avoiding serious sequels of further instability and in order to allow early mobilization and rehabilitation, a second operation for stabilization is mandatory. For this purpose, occipitocervical instrumentation and fusion are recommended by most authors [55–60].

Coincidence of os odontoideum in achondroplastic patient and possibility of cervicomedullary compromise due to augmented effect of these two pathologies is an extremely rare entity revealing only five children with this combination in a very careful review of the literature [17–20]. Therefore, the current case is the first example of os odontoideum discovered in an adult with achondroplasia.

When a surgeon is faced with combination of these two pathologies attempts should be directed toward the management of both [17–20]. This means that reduction and stabilization of atlantoaxial dislocation alone may fail to ensure an adequate decompression. Therefore, the procedure of decompression will be only completed when suboccipital craniectomy, C1 arch laminectomy, and removal of the tight fibrous bands are accomplished.

The outcome before establishment of myelopathy is good in both instances in isolation where delay in diagnosis and surgery in myelopathic patients will require a longstanding course of physiotherapy and patience although some deficits might remain irreversible. Undoubtedly, prognosis in the presence of both pathologies is worse than each disease in isolation.

In summary, presence of os odontoideum in achondroplasia is extremely rare event in particular in adults. Dynamic cervical radiographs and axial and reformatted CT as well as MRI are required for early detection of this coexistence. In coexistence of these two pathologies, instability due to os odontoideum can traumatize the already compromised cervicomedullary neural tissues. For adequate decompression of the cervicomedullary region, both pathologies should be surgically targeted. Eventually, surgical management will be more challenging than any of them in isolation; the strategy should include reduction and stabilization of atlantoaxial dislocation with subsequent small suboccipital craniectomy, C1 arch laminectomy, and removal of the dense fibrous bands. Degree of recovery depends on the neurological status at the time of surgery. Obviously, in those with disabling quadriparesis chance of good recovery is very low.
